# A Post-Lockdown Assessment of Albendazole Treatment Coverage in Mass Drug Administration Campaigns Implemented Before and During COVID-19 Pandemic in Ekiti, Southwest Nigeria

**DOI:** 10.3389/ijph.2023.1605510

**Published:** 2023-02-09

**Authors:** Hammed O. Mogaji, Hilary I. Okoh, Abiodun M. Lawal, Kayode H. Ojo, Ayodele J. Marcus, Nwana O. Aaron, Damilola R. Adeleye, Francisca O. Olamiju, Uwem F. Ekpo

**Affiliations:** ^1^ Parasitology and Epidemiology Unit, Department of Animal and Environmental Biology, Federal University Oye-Ekiti, Oye, Nigeria; ^2^ Clinical Psychology Unit, Department of Psychology, Federal University Oye-Ekiti, Oye, Nigeria; ^3^ Neglected Tropical Diseases Unit, Ekiti State Primary Healthcare and Development Agency, Ado Ekiti, Nigeria; ^4^ Mission to Save the Helpless (MITOSATH), Jos, Nigeria; ^5^ Parasitology and Epidemiology Unit, Department of Pure and Applied Zoology, Federal University of Agriculture, Abeokuta, Abeokuta, Nigeria; ^6^ Bioscience Research Programme, Institute of Food Security, Environmental Resource and Agricultural Research (IFSERAR), Federal University of Agriculture, Abeokuta, Nigeria

**Keywords:** COVID-19, Nigeria, soil-transmitted helminthiasis, albendazole, mass drug administration

## Abstract

**Objective:** This study assessed the coverage of albendazole (ALB) in mass drug administration (MDA) programs implemented before (2019) and during the (2020 and 2021) COVID-19 pandemic in Ekiti State, Nigeria.

**Methods:** Standardized questionnaires were administered to 1,127 children across three peri-urban communities to ascertain if they received and swallowed ALB across the years. Reasons, why ALB was not received, were documented and analyzed in SPSS. 20.0.

**Results:** In 2019, the medicine reach was between 42.2%–57.8%, however, during the pandemic, the reach significantly reduced to 12.3%–18.6%, and increased to 28.5%–35.2% in 2021 (*p* < 0.000). About 19.6%–27.2% of the participants have missed 1 MDA, while 26.9%–37.8% and 22.4%–32.8% have missed 2 and 3 MDAs, respectively. The majority who did not receive ALB (60.8%–75%) claimed drug distributors never came, while about 14.9%–20.3% mentioned they did not hear about MDA. However, individual compliance towards swallowing was above 94% across the study years (*p* < 0.00).

**Conclusion:** These results highlight the need to explore the perceptions of those who have consistently missed MDAs, and also understand the health-system-related issues including those imposed by the pandemic affecting MDA.

## Introduction

Soil-Transmitted Helminthiasis (STH) is one of the most important neglected tropical diseases (NTDs) in sub-Saharan Africa (SSA) [1]. The global burden of STH is enormous, with over a billion people at risk, about 6300 deaths and 3.5 million disability-adjusted life years (DALYs) [2]. The disease is common in areas where water, sanitation and hygiene resources are limited, with school-aged children (SAC) between ages 5 and 14 years constituting the most vulnerable group, due to their increased mobility, developing immunity and poor hygiene practices [3, 4]. The cornerstone strategy for STH control has been through periodic mass administration of albendazole medicines (MDA) to SAC through teachers or community volunteers in schools and communities, respectively [3].

WHO recommends consistent MDA with albendazole to at least 75% of at-risk populations in endemic countries to achieve elimination [3] and has coordinated the annual distribution of over 400 million albendazoles to treat about 576.4 million children in 2019 [2, 5]. Successful MDA requires community participation and health workers’ commitment [6]. In areas with poor treatment coverage, and where a large segment of SAC is consistently not included or refuses to participate in MDA, a potential parasite reservoir is left untreated, thus risking continued transmission [7–9]. Research targeted at identifying these individuals, and understanding the individual, social, cultural, and health-system factors that interfere with MDA coverage is therefore important. Furthermore, with the advent of the COVID-19 pandemic, there have been severe disruptions in routine public health services [10], with concerns that accompanied shifts in policies such as restriction of movement, closure of schools and delay in MDA during the pandemic might have a negative impact on MDA implementation [11].

Nigeria shares over 25% of the NTDs burden in SSA, including those caused by STH. Since 2014, endemic States in the country have benefitted from the MDA program targeted at STH [12]. However, in 2020, MDA campaigns were temporarily suspended following the lockdown orders instituted by the government across all states in response to the COVID-19 pandemic [13, 14]. In October 2020, lockdown orders were lifted, and MDA resumed following standard operating guidelines stipulated by WHO [10]. MDAs were prioritized in selected states across the country. Ekiti was among the states to resume MDA with support from the state government and a non-governmental organization, Mission to Save The Helpless (MITOSATH). The state is located in the southwestern part of the country and has 16 local government areas (LGAs). Before the pandemic, there were speculations that a large reservoir of children who have consistently missed MDAs exist in some communities in Ikere, one of the peri-urban LGAs in the State. This speculation was reinforced by a recent analysis of MDA programmatic data in the LGA. This study, therefore, evaluated the coverage of albendazole medicines in MDA implemented before and during the pandemic (2019–2021) in Ogbonjanna, Okekere and Okeosun communities. In addition, the study evaluated the proportion of SAC that have consistently missed MDAs and established factors associated with their non-participation or refusals. This study was part of a larger implementation study aimed at understanding the effect of the COVID-19 pandemic on MDA campaigns, to support the design and implementation of context-fitting strategies to strengthen MDA.

## Methods

### Study Area

Ekiti is one of the rural and NTD endemic states in the southwestern part of Nigeria. The state has 16 LGAs, with its capital located in Ado-Ekiti. The majority of the LGAs are rural (*n* = 13), followed by peri-urban (*n* = 2) and urban LGA (*n* = 1). An estimated population of 2.2 million people live within the state, with an agrarian population mostly in rural communities. Ikere LGA is one of the peri-urban LGAs, with great proximity to the capital city. The MDA program targeted at STH commenced in the LGA in 2015, with a biennial mode of operation.

### Study Design, Selection of Communities and Sample Size Estimations

This study employed a cross-sectional sampling design involving questionnaire administration and household visitations across three communities in Ikere LGA i.e., Ogbonjana, Oke-Osun and Okekere. These communities were purposively selected based on their poor MDA coverage in 2019 (<75%). The methodology employed for estimating sample size followed the guidelines provided by WHO for coverage evaluation surveys with some modifications (WHO, 2016). As an initial step, the total population of school-aged children in each community was extracted from the 2022 updated village census register obtained from the NTDs control department. Sample size was calculated using the formula; $n_{s}=\frac{n}{1+\left(n/N\right)}$ and $n=\frac{z^{2}p\left(1-p\right)}{d^{2}\left(1-r\right)}$ , where n_s_ is the required sample size, N is the target population size, p is the proportion of reported therapeutic coverage in 2019 i.e., 60.2%, d is the relative precision of 5%, r is the non-response rate from participants, and Z is the 1.96 z-score that corresponds to 95% confidence limit. Based on WHO recommendations, sample size estimates were optimized by underestimating p, using the formula *p* = $\left(Reported\;therapeutic\;coverage-15\right)\%$ (WHO, 2016). The minimum sample size determined for Ogbonjana, Oke-Osun and Okekere communities were 343, 424, 399, respectively (Table 1).

**TABLE 1:** Sample size estimation across selected study communities. Nigeria, 2019–2021.

Communities	Total Pop.	SAC Pop.	No. of HH	No. of Seg.	HH per Seg.	Sample size	SAC per Seg.	HH to be visited per Seg
Ogbonjana	12,548	3,513	2,510	31	80	343	11	5
Oke-Osun	28,458	7,698	5,692	71	80	424	6	3
Okekere	13,060	3,657	2,612	33	80	399	12	6

Pop, population; SAC, school-aged children; HH, household; Seg, segments.

### Estimation of Household Size, Segments, and Sample Size Per Segment

The WHO coverage evaluation survey guide was adopted in estimating household size and segmenting the communities [15]. In brief, the average number of households in each community was estimated using the formula; Average number of household = $\frac{Population\;of\;community}{5}$ . While the number of households segments within each community was estimated using the formula; HH segments per community = $\frac{Number\;of\;households\;in\;a\;community}{80}$ . In each community, the leaders and representatives were mobilized through local consultations with the research team members to divide the community into pre-determined number of segments (Table 1). Boundaries such as roads, markets, rivers were taken into consideration during such segmentation. While assuming that community residents are equidistantly distributed in each community, the estimated sample size for each identified segment was determined using the formula; Sample size of SAC per segment = $\frac{Estimated\;SAC\;sample\;size\;in\;the\;community}{number\;of\;segments}$ . Based on the assumption that 2 out of 5 members of a household are SAC, the estimated number of households visited was calculated using the formula; HH visited per community = $\frac{Sample\;size\;of\;SAC\;per\;segment}{2}$ . These estimates guided field researchers of the minimum number of household and SAC recruited in each of the identified segment of the community (Table 1).

### Enumeration of Households and Selection of Study Participants

The survey team, with the help of a local guide visited each segment to identify a common walking path. All households along the identified walk path were enumerated and labelled using colored crayons. Households were marked sequentially, starting from 1 to n, irrespective of the condition of the house. A systematic sampling method was then employed to select households to be recruited in the study. A sampling interval (K) was determined using the formula; K = $\frac{Estimated\;HH\;per\;segment}{Estimated\;HH\;to\;be\;visited}$ (Table 1). Following the estimation of the sampling interval, a random number (r) between 1 and K was selected using paper balloting. The number selected (r) corresponds to the first household to be visited in the segment. The second house to be selected (r2) was determined using the formula r2 = K + r. Subsequent households were selected following this pattern (for instance: r3 = K + r2; r4 = K + r3), until the estimated number of households to be visited is met. If a selected household was inaccessible or abandoned, the next household was selected as a replacement. Each household was given a unique ID comprising of the code for the state, LGA, community, segment, and household (Supplementary Material S1). In each of the selected household, a household head was identified, and the purpose of the visit was explained before seeking consents. Only eligible school-aged children who are between 5 and 14 years, and their parents/guardians were invited to participate in the study.

### Ethical Considerations

This study received ethical approval from Ekiti State Ethical Review Board (MOH/EKHREC/EA/P/33). Permissions and consents were also obtained from the NTD control unit in the state. Prior commencement of household visitation, consent was sought from the community leaders in each of the selected communities. Informed assent was also obtained from all children in addition to another written consent provided by their parent or legal guardian. In cases where the parents or legal guardian were not physically present, verbal consent through telephone were obtained. Unique identifiers and a password protected database were used to protect personal information of the study participant (Supplementary Material S2).

### Data Collection and Tools

Data were collected using WHO standardized coverage evaluation survey questionnaire (Supplementary Material S3) designed into electronic forms and administered by a team of 8 research assistants (in a pair of 4). Each pair comprises of an interviewer and an assistant and was accompanied by a local guide during the period of the survey. The standardized questionnaire was used to collect information on demographics, program reach and coverage across the MDAs implemented before the pandemic (2019) and during the pandemic (2020 and 2021). Questions included were 1) if SAC were offered the medicine (program reach), 2) if SAC swallowed the medicine (survey coverage), and 3) reasons why medicines were either not offered and/or swallowed. SAC was shown the samples of the medicine administered to facilitate recall during the interviews. Interviews were also conducted in Yoruba and English languages (as appropriate). However, in areas where the targeted respondents were unavoidably absent, or too young to respond, the household head responded on their behalf.

### Data Management and Analysis

Data collected were downloaded from cloud and imported into SPSS 20.0 statistical software (SPSS, United States) for analysis. Descriptive statistics such as frequencies, proportions, means, and standard deviations were used to summarize and present the proportions on background and socio-demographic characteristics of the respondents. Only children between age 5 and 14 across the survey years were considered eligible. As such, data of children below age 8 for year 2019, age 7 for year 2020 and age 6 for year 2021 were excluded from the analysis. Two percentage metrics; the self-reported “survey coverage,” which is the percentage of the eligible population who swallowed the medicine administered, and the “program reach,” which is the percentage of respondents who were offered the drug were estimated. The following formulas were used to calculate the survey coverage and program reach, respectively; Survey coverage = $\frac{Number\;of\;individuals\;who\;swallowed\;the\;drug}{Total\;number\;of\;individuals\;surveyed}$ and Program reach = $\frac{Number\;of\;individuals\;who\;were\;offered\;the\;drug}{Total\;number\;of\;individuals\;surveyed}$ . Individual compliance with MDA treatment was identified by comparing the survey coverage to the program reach. Pearson chi-square/Fisher’s exact test was used to assess if there were significant differences in programme reach and survey coverage before the pandemic and during the pandemic. Significant level was set at 5%.

## Results

### Demographic Characteristics of Study Participants

A total of 1,127 school-aged children across 625 households were recruited, with 736 (65.3%) of them being eligible for MDA in 2019, 881 (78.2%) in 2020 and 996 (88.4%) in 2021. The mean age of the respondents ranges from 9.48 ± 2.56 to 10.82 ± 2.00, and by gender there were no significant differences in the proportion of males or females recruited (*p* > 0.05) (Table 2).

**TABLE 2:** Demographic characteristics of the study population. Nigeria, 2019–2021.

	Communities	Households recruited	Participants recruited	Eligible participants^a^	Female	Male	p-value (gender)	Age (Mean + S.D)
2019	Ogbonjanna	188	319	225 (70.5)	122 (54.2)	103 (45.8)	0.36	10.82 ± 2.00
	Okekere	182	322	205 (63.7)	105 (51.2)	100 (45.8)		10.70 ± 1.96
	Okeosun	255	486	306 (62.9)	147 (48.0)	159 (52.0)		10.71 ± 2.00
	Total	625	1,127	736 (65.3)	374 (50.8)	362 (49.2)		10.72 ± 1.99
2020	Ogbonjanna	188	319	266 (83.4)	141 (53.0)	125 (47.0)	0.23	10.23 ± 2.30
	Okekere	182	322	242 (75.2)	125 (51.7)	117 (48.3)		10.14 ± 2.24
	Okeosun	255	486	373 (76.7)	174 (46.6)	199 (53.4)		10.04 ± 2.31
	Total	625	1,127	881 (78.2)	440 (49.9)	441 (50.1)		10.12 ± 2.29
2021	Ogbonjanna	188	319	288 (90.3)	149 (51.7)	139 (48.3)	0.32	9.91 ± 2.48
	Okekere	182	322	288 (89.4)	143 (49.7)	145 (50.3)		9.48 ± 2.56
	Okeosun	255	486	420 (86.4)	194 (46.2)	226 (53.8)		9.59 ± 2.52
	Total	625	1,127	996 (88.4)	486 (48.6)	510 (51.2)		9.65 ± 2.52

N: Number of respondents interviewed.

^a^ Only children between age 5 and 14 across the survey years were considered eligible. As such, children below age 8 for year 2019, age 7 for year 2020 and age 6 for year 2021 were excluded from the analysis.

### Program Reach and Coverage Across the Study Communities (2019–2021)

**FIGURE 1:**
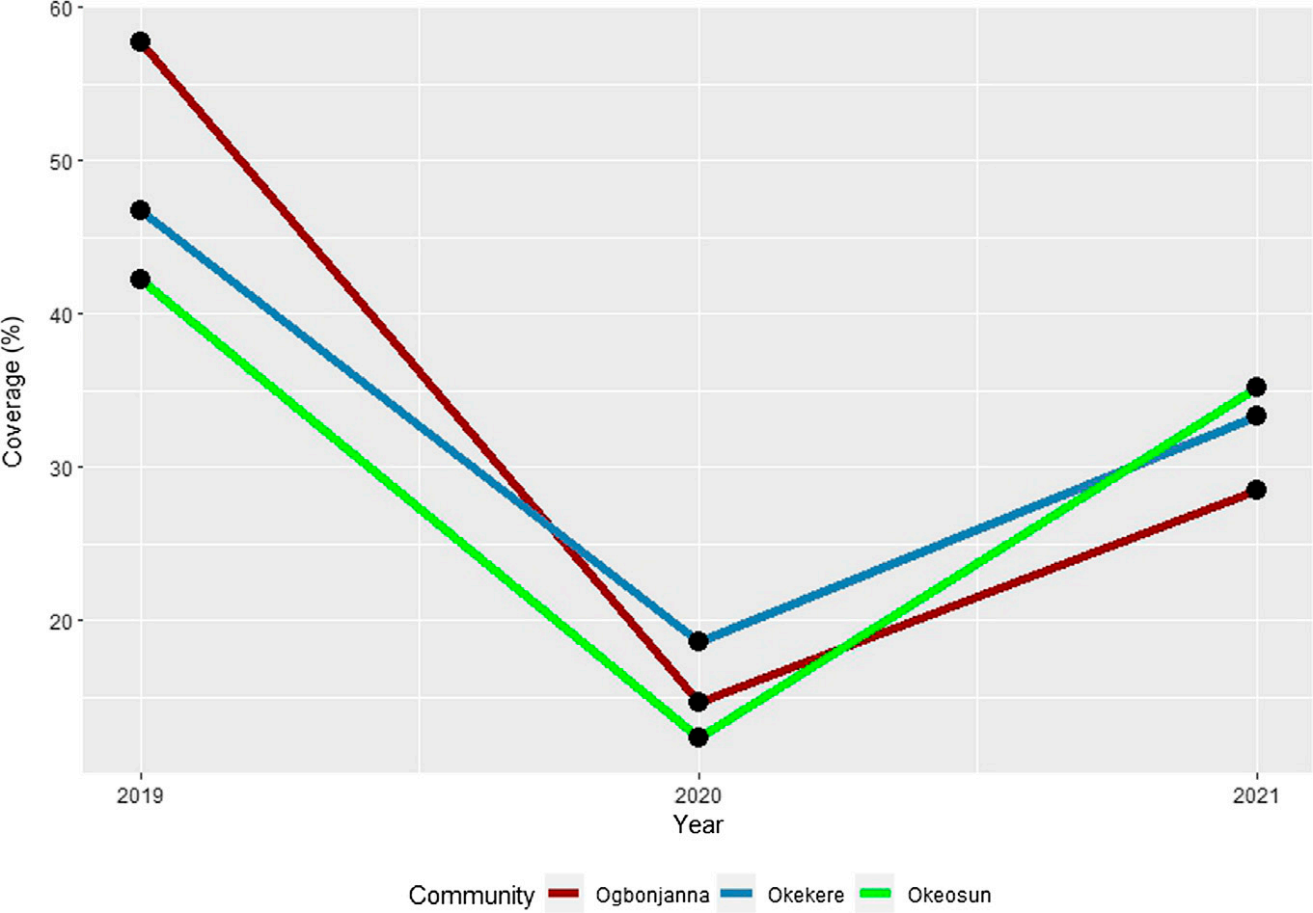
Program reach across the study communities. Nigeria, 2019–2021.

In Ogbonjana, about 57.8% (130/225) of those interviewed in 2019 were offered albendazole medicines, and compliance towards swallowing was 94.6%. However, in 2020, only 14.7% (39/266) were offered, with individual compliance of 92.3%. Furthermore in 2021, only 28.5% (82/288) were offered, with compliance of 92.6%. There were significant differences in the proportions of those offered albendazole across the study years (*p* = 0.000), with significant decline in 2020. However, there were no significant differences in the proportions of those swallowing albendazole across the study years (*p* = 0.999) (Table 3; Figure 1).

**TABLE 3:** Program reach and coverage across the study communities. Nigeria, 2019–2021.

Communities	Indicators	2019	2020	2021	p-value	p-value
		ALB	ALB	ALB	Offered vs. Not offered	Offered vs. Swallowed
Ogbonjanna	Persons recruited	225	266	288		
	Persons who were offered	130 (57.8)	39 (14.7)	82 (28.5)	0.000	0.999
	Persons who were not offered	80 (35.6)	208 (78.2)	177 (61.5)		
	Persons who swallowed	123 (54.7)	36 (13.5)	76 (26.4)		
	Persons who did not swallow	6 (2.7)	2 (0.8)	6 (2.1)		
	Individual compliance (%)	94.6	92.3	92.6		
Okekere	Persons recruited	205	242	288		
	Persons who were offered	96 (46.8)	45 (18.6)	96 (33.3)	0.000	0.999
	Persons who were not offered	107 (52.2)	193 (79.8)	190 (66.0)		
	Persons who swallowed	91 (44.4)	43 (17.8)	91 (31.6)		
	Persons who did not swallow	5 (2.4)	2 (0.8)	4 (1.4)		
	Individual compliance (%)	94.8	95.6	94.8		
Okeosun	Persons recruited	306	373	420		
	Persons who were offered	129 (42.2)	46 (12.3)	148 (35.2)	0.000	0.971
	Persons who were not offered	168 (54.9)	317 (85.0)	269 (64.0)		
	Persons who swallowed	123 (40.2)	45 (12.1)	147 (35.0)		
	Persons who did not swallow	6 (2.0)	1 (0.3)	1 (0.2)		
	Individual compliance (%)	95.3	97.8	99.3		
Summary	Persons recruited	736	881	996		
	Persons who were offered	355 (48.2)	130 (14.8)	326 (32.7)	0.000	0.991
	Persons who were not offered	355(48.2)	718 (81.5)	636 (63.9)		
	Persons who swallowed	337 (45.8)	124 (14.1)	314 (31.5)		
	Persons who did not swallow	17 (2.3)	5 (0.6)	11 (1.1)		
	Individual compliance (%)	94.9	95.4	96.3		

ALB, albendazole.

Similarly, in Okekere, about 46.8% (96/205) of those interviewed in 2019 were offered albendazole medicines, and compliance towards swallowing was 94.8%. However, in 2020, only 18.6% (45/242) were offered, with individual compliance of 95.6%. Furthermore in 2021, only 33.3% (96/288) were offered, with compliance of 94.8%. There were significant differences in the proportions of those offered albendazole across the study years (*p* = 0.000), with significant decline in 2020. However, there were no significant differences in the proportions of those swallowing albendazole across the study years (*p* = 0.999) (Table 3; Figure 1).

In Oke-osun, about 42.2% (129/306) of those interviewed in 2019 were offered albendazole medicines, and compliance towards swallowing was 95.3%. However, in 2020, only 12.3% (46/373) were offered, with individual compliance of 97.8%. Furthermore in 2021, only 35.2% (148/420) were offered, with compliance of 99.3%. There were also significant differences in the proportions of those offered albendazole across the study years (*p* = 0.000), with significant decline in 2020. However, there were no significant differences in the proportions of those swallowing albendazole across the study years (*p* = 0.971) (Table 3; Figure 1).

### Profile of Participants Based on Participation in MDA Programs Across the Study Communities (2019–2021)

About 19.6%–27.2% of the participants have missed 1 MDA, while 26.9%–37.8% and 22.4%–32.8% have missed 2 and 3 MDAs, respectively. By communities, majority of the study participants in Ogbonjana (108, 37.8%) and Okekere (90, 31.9%) had missed 2 MDAs. However, in Oke-osun, majority had missed 3 MDAs (137, 32.9%) (Table 4).

**TABLE 4:** Profile of participants based on participation in MDA programs across the study communities. Nigeria, 2019–2021.

Communities	NR	Ineligible*	Number of MDA missed		
			One	Two	Three
Ogbonjanna	286^a^	58 (20.3)	56 (19.6)	108 (37.8)	64 (22.4)
Okekere	282^b^	43 (15.2)	71 (25.2)	90 (31.9)	78 (27.7)
Okeosun	416^c^	54 (13.0)	113 (27.2)	112 (26.9)	137 (32.9)
Total	984	155 (15.8)	240 (24.4)	310 (31.5)	279 (28.4)

^a,b,c^ Data for 2, 6, and 4 participants are missing; NR: Number recruited.

*Respondents who were sick during the MDA, were considered ineligible.

### Reasons Why Albendazoles Were Not Received Across the Study Years (2019–2021)

Of the 355 participants recruited before the pandemic, majority of them (216, 60.8%) mentioned that nobody (drug distributors) came to their house, while others (72, 20.3%) mentioned that they did not hear about MDA. Similarly, during the pandemic, more participants mentioned that nobody (drug distributors) came to their house, 534(74.4%) in 2020 and 477(75.0%) in 2021. However, the proportion of those who did not hear about MDA reduced during the pandemic, 123(17.1%) in 2020 and 95(14.9%) in 2021 (Table 5).

**TABLE 5:** Reasons why albendazole medicines were not received across the study years. Nigeria, 2019–2021.

Reasons	2019 (N = 355)	2020 (N = 718)	2021 (N = 636)	p-value
Nobody came	216 (60.8)	534 (74.4)	477 (75.0)	0.000
Did not hear about MDA	72 (20.3)	123 (17.1)	95 (14.9)	
Absent	15 (4.2)	188 (2.5)	22 (3.5)	
Parent disapproved/rejected	17 (4.9)	14 (2.0)	21 (3.3)	
No reasons	10 (2.8)	2 (0.3)	0 (0)	
Drugs ran out	3 (0.8)	2 (0.3)	2 (0.3)	

## Discussion

The advent of COVID-19 pandemic in 2020 disrupted routine public health service including implementation of mass drug administration (MDA) campaigns. In the year of the pandemic (2020–2021), school-based distribution of medicines was disallowed, and MDA for albendazole were implemented using community drug distributors as volunteers. In this study, we evaluated the coverage of albendazole (ALB) medicines in MDA programs implemented before and during the pandemic, as part of a larger study targeted at understanding the effect of pandemic on implementation of MDA programs. Our primary aim was to validate existing speculations that school-aged children have consistently missed MDAs, and secondarily, investigate if there were significant reductions in treatment coverage during the pandemic, and identify factors associated with such reductions. Following WHO recommended guidelines, we measured program reach as the proportion of individual who were offered/received ALB medicines [15], and our findings revealed unsatisfactory rates below the 75% threshold. The program reach was higher in 2019, compared to the pandemic years, and this trend was common across all the study communities. Our findings validated existing speculations as almost half of the eligible children interviewed had missed MDA in the year before the pandemic. The situation worsened during the pandemic with about 82% and 64% of them missing MDAs in 2020 and 2021, respectively. These findings are similar with the coverage report for MDA implemented during the pandemic in Guinea, with reservations that coverage was below what would have been expected under non-pandemic circumstances [16].

The degree of non-participation/compliance in MDA programs, and transmission intensity of parasites are important indicators in elimination programs. Whenever, transmission intensity is high, a consistently high treatment coverage (≥75%) will be required over at least 5 years to move towards transmission elimination [17]. As such, the high non-participation in MDA programs reported in this study is a critical issue that requires attention [7–9]. At the moment, MDA participation patterns are particularly understudied [18], as it is difficult to identify individual non-compliers after MDA programs. There are propositions to implement longitudinal components that document MDA participation patterns in monitoring and evaluation programs [17], however, the feasibility of this idea is yet to be assessed, as MDA programs have funding limitations, and combining a longitudinal component would incur additional costs beyond budget limits [17]. More importantly, such follow-up component would require a more refined data collection and curation methodology, which are currently missing. In addition, successive visits by distributors to administer medicines to those who has repeatedly missed annual treatment, will often be prohibitively time-consuming and hence costly [17]. It is therefore important to leverage on technological innovations incorporated into existing MDA implementation processes, to better document who is treated and when treatment was made, as well as who is not treated, and why treatment was not made.

To this end, our study, retrospectively analyzed data from children in three communities, across three implementation years. Although compliance to swallowing the medicines were high, we however, observed that about one-third of the population had missed 1, 2 and 3 MDAs, respectively. These persons were systematically selected and recruited for follow-up studies, particularly on factors associated with non-participation. Reasons attributed to non-participation were non-visitation of community volunteers, absenteeism of household members during MDA and not having enough information about the medicines. These reasons were frequently reported during the pandemic years and are consistent with other existing reports from India [19], Kenya [20], Ethiopia [21], Liberia [22] and Mali [23]. It is therefore important to reflect on these gaps and identify factors that might contribute to non-visitation of community volunteers and absenteeism of household members especially during the post-COVID lockdown era [11]. For instance, drug shortages, lack of motivation for the volunteers, poor timing of interventions might contribute to inability of drug distributors to visit the households, or even meet household members at home [22, 24]. It is therefore important to further invest efforts in understanding factors associated with non-visitation of community volunteers and absenteeism of household members during MDAs. These findings would be useful in optimizing participation and strengthening MDA delivery [25].

### Conclusion

Our findings validate existing speculations that a group of eligible school-aged children had consistently missed MDAs targeted at controlling STH. The program reach was significantly lower during the pandemic compared to the year before the pandemic, with reasons attributed to non-visitation of community volunteers, absenteeism of household members and not having enough information about the medicines. It is therefore important to further explore perceptions of those who have consistently missed MDAs, and also understand how health-system related issues including those imposed by the pandemic have affected MDA.
