# Supplementary material for: A Post-Lockdown Assessment of Albendazole Treatment Coverage in Mass Drug Administration Campaigns Implemented Before and During COVID-19 Pandemic in Ekiti, Southwest Nigeria
Source: Int J Public Health. 2023 Feb 9;68:1605510. doi: 10.3389/ijph.2023.1605510 (PMC9948738; doi:10.3389/ijph.2023.1605510)
Supplement: Supplementary file 2 [file DataSheet3.docx]

**S3 : WHO standardized questionnaire tool**

**Household Survey to Measure Coverage of [*list drug(s) here*] Among [*list survey population here* ]**

Interviewer Code: |__|__|__| District: |__________________| Sub-district: |_______________|

Subunit (EA/Village/Other_______________): |___________________________| HH No: |__|__|__|

Date of interview (dd/mm/yyyy): |__|__|/|__|__|/|__|__|__|__| Household Head:_______________________________

Survey population: ________________________________________________

|  | | | | | ***[Drug 1]*** | | | | | ***[Drug 2]*** | | | | |
| --- | --- | --- | --- | --- | --- | --- | --- | --- | --- | --- | --- | --- | --- | --- |
| # | First Name | Sex (M/F) | Age  (Years) | HH member present (Y/N) | Offered the drug(s)? (Y/N/DK) | Reason not offered^a^ | Swallow the drug(s)? (Y/N/DK) | Reason DID NOT swallow^b^ | Reason DID swallow^c^ | Offered the drug(s)? (Y/N/DK) | Reason not offered^a^ | Swallow the drug(s)? (Y/N/DK) | Reason DID NOT swallow^b^ | Reason DID swallow^c^ |
| 1 |  |  |  |  |  |  |  |  |  |  |  |  |  |  |
| 2 |  |  |  |  |  |  |  |  |  |  |  |  |  |  |
| 3 |  |  |  |  |  |  |  |  |  |  |  |  |  |  |
|  |  |  |  |  |  |  |  |  |  |  |  |  |  |  |
|  |  |  |  |  |  |  |  |  |  |  |  |  |  |  |
|  |  |  |  |  |  |  |  |  |  |  |  |  |  |  |
|  |  |  |  |  |  |  |  |  |  |  |  |  |  |  |
|  |  |  |  |  |  |  |  |  |  |  |  |  |  |  |

| ^a^Reason treatment was not offered | | | | ^b^Reason treatment *was not* swallowed | | ^c^Reason treatment *was* swallowed |
| --- | --- | --- | --- | --- | --- | --- |
| 1= | Underage | 6= | Didn't hear about MDA | 1= | Fear of side effects | 1= Fear of disease(s) |
| 2= | Pregnant | 7= | Drug ran out | 2= | Bad taste | 2=To treat disease(s) |
| 3= | Breast feeding | 8= | Nobody came | 3= | Not sick | 3= Because it was given/ free |
| 4= | Sick | 9= | Other | 4= | Not enough information given | 4=Useful information from CDD |
| 5= | Absent |  |  | 5= | Other | 5=Other |

Other independent variables such as sex, age, ethnicity, marital status, religion, household income, occupation, phone number and educational status of the parent or household head will be documented

Y=”Yes”, N=”No”, DK = “Don’t Know”
