# Supplementary material for: A Post-Lockdown Assessment of Albendazole Treatment Coverage in Mass Drug Administration Campaigns Implemented Before and During COVID-19 Pandemic in Ekiti, Southwest Nigeria
Source: Int J Public Health. 2023 Feb 9;68:1605510. doi: 10.3389/ijph.2023.1605510 (PMC9948738; doi:10.3389/ijph.2023.1605510)
Supplement: Supplementary file 3 [file DataSheet2.docx]

**S2: Informed consent form**

### **Participant Information Sheet**

**Name of Project:** Impact of COVID-19 on mass drug administration campaigns targeting schistosomiasis and soil-transmitted helminthiasis: A post-lockdown evaluation of coverage, non-compliance and health workers motivation in some rural communities in Southwest Nigeria.

**Name of Principal Investigator:** Dr Mogaji Hammed

**Name of Organization:** Federal University Oye-Ekiti, Federal University of Agriculture Abeokuta, Ekiti State SPHCDA and MITOSATH

**Funding Source:** African Regional Network on Neglected Tropical Diseases (ARNTD)

**Objectives of the study:**

I will like to read to you, the information contained in this paper, about a study we would like to do with you and your family, here in your community. We are researchers who work at a university. We are conducting this study about the medicines that are usually distributed in schools and the community. We are trying to ask some few questions from children, parents and health workers about these medicines. We plan to use the information we get, to improve the way the medicines are delivered. Your participation in this study will help us understand how to deliver the medicines better in this community. After reading the information in this document to you, we will like you to take a decision if you will be part of the study, or not. If so, you will be asked to sign the last page of this consent form to confirm your willingness to participate.

**Participant Selection**

You are being invited to participate in this research because of your importance in the community.

**Voluntary Participation**

Your participation in this study is entirely voluntary. It is your choice whether you want to participate or not.

**Risks**

If you feel uncomfortable at any point in the interview, you do not have to continue, if you don't wish to do so. Also, if there are some questions you do not wish to be asked, or answer, kindly discuss this with us.

**Benefits**

There will be no direct benefit to you, but your participation is likely to provide information on how to improve the delivery of medicines following COVID 19.

**Confidentiality**

If it is ok with you, we would like to take notes and audio record the interviews. The information that we collect from these activities will be anonymized and stored securely in a confidential manner. They will also be deleted after analysis.

**Sharing the results**

The knowledge obtained from this research will be shared with the community before it is made available to the public. We will share the findings for the wider public in conferences, journals and meetings so that other interested people may learn from the research.

**Who to contact**

If you have any questions, you can ask them now or later. If you wish to ask questions or make a complaint about the research, you may contact the Lead Researcher: Dr Mogaji Hammed Oladeji ([mogajihammed@gmail.com](mailto:mogajihammed@gmail.com), +2348156018982)

**Consent:** I heard and understood the reading and explanations. My questions have been answered. I willingly agree to participate.

**Name of the Participant**:

Study Participant Signature Date Time


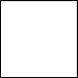
 Study Participant's Fingerprint

I heard and understood this document. My questions have been answered. I voluntarily agree that the participant of whom I am a parent or legal guardian participates:

**Name of legal guardian:**

Signature of legal guardian Date Time

**Name of researcher taking the consent**:

Investigator’s Signature Date Time
