# Supplementary material for: A Post-Lockdown Assessment of Albendazole Treatment Coverage in Mass Drug Administration Campaigns Implemented Before and During COVID-19 Pandemic in Ekiti, Southwest Nigeria
Source: Int J Public Health. 2023 Feb 9;68:1605510. doi: 10.3389/ijph.2023.1605510 (PMC9948738; doi:10.3389/ijph.2023.1605510)
Supplement: Supplementary file 4 [file DataSheet1.docx]

**S1 : Identification code for households and participants**

| **ITEMS** | **Code** | **Name** |
| --- | --- | --- |
| State | 6 | Ekiti |
| LGA | 1 | Ikere |
| Community 1 | 01 | Ogbonjana |
| Community 2 | 02 | Oke-Osun |
| Community 3 | 03 | Okekere |
| Segments | S1-S100 | A number represents a segment which is a group of 50 houses |
| Separator | [dot] | A dot to separate the segment from the household |
| Households | H1-H100 | A number represents a household which is a group of 5 persons |
| Separator | [dot] | A dot to separate the household from participant |
| Participant | P1-P1000 | A number represent a participant |
| Teacher | T1-T2 | A number represent a teacher |
| CDDs | C1-C2 | A number represent a CDDs |
| FLHW | F1-F2 | A number represent a FLHW |
| Household ID | 6101S5.H4 | Ekiti, Ikere, Ogbonjanna, Segment 5, Household 4 |
| Participant ID | 6101S5.H4.P20 | Ekiti, Ikere, Ogbonjanna, Segment 5, Household 4, Participant 20 |
| CDD ID | 6101.C1 | Ekiti, Ikere, Ogbonjanna, CDD1 |
| Teacher ID | 6101.T2 | Ekiti, Ikere, Ogbonjanna, Teacher 1 |
| FLHW | 6101.F1 | Ekiti, Ikere, Ogbonjanna, FLHW 1 |
